# Effectiveness and Safety of Apixaban vs. Warfarin in Venous Thromboembolism Patients with Obesity and Morbid Obesity

**DOI:** 10.3390/jcm10020200

**Published:** 2021-01-08

**Authors:** Alexander Cohen, Janvi Sah, Theodore Lee, Lisa Rosenblatt, Patrick Hlavacek, Birol Emir, Allison Keshishian, Huseyin Yuce, Xuemei Luo

**Affiliations:** 1 Department of Hematological Medicine, Guy’s & St Thomas’ NHS Foundation Trust, King’s College London, Westminster Bridge Road, London SE1 7EH, UK; 2 STATinMED Research, Ann Arbor, MI 48108, USA; jsah@statinmed.com (J.S.); akeshishian@statinmed.com (A.K.); 3 Pfizer Inc., New York, NY 10017, USA; theodore.lee@pfizer.com (T.L.); patrick.hlavacek@pfizer.com (P.H.); birol.emir@pfizer.com (B.E.); 4 Bristol Myers Squibb Company, Lawrenceville, NJ 08648, USA; lisa.rosenblatt@bms.com; 5 New York City College of Technology, City University of New York, New York, NY 11201, USA; HYuce@citytech.cuny.edu; 6 Pfizer Inc., Groton, CT 06340, USA; Xuemei.Luo@pfizer.com

**Keywords:** venous thromboembolism, direct oral anticoagulants, apixaban, warfarin, recurrent venous thromboembolism, major bleeding, obesity, morbidly obese

## Abstract

This study integrated 5 United States healthcare claims databases to evaluate the risk of recurrent venous thromboembolism (VTE) and major bleeding (MB) among VTE patients who initiated apixaban vs. warfarin, stratified by obesity. Obese and morbidly obese patients were identified based on diagnosis codes. Stabilized inverse probability treatment weighting (IPTW) was conducted to balance observed patient characteristics between treatment cohorts. An interaction analysis was conducted to evaluate treatment effects of apixaban vs. warfarin according to obesity status. Cox proportional hazard models were used to evaluate the risk of recurrent VTE and MB among IPTW weighted obese and morbidly obese patients. A total of 112,024 non-obese patients and 43,095 obese patients were identified, of whom 19,751 were morbidly obese. When stratified by obesity status post-IPTW, no significant interactions were observed for effects of apixaban vs. warfarin on recurrent VTE or MB (interaction *p* > 0.10). Among IPTW obese and morbidly obese patients, apixaban was associated with a significantly lower risk of recurrent VTE (obese: 0.73 [0.64–0.84]; morbidly obese: 0.65 [0.53–0.80]) and MB (obese: 0.73 [0.62–0.85]; morbidly obese: 0.68 [0.54–0.86]) as compared with warfarin. In this large sample of obese and morbidly obese VTE patients, apixaban had a significantly lower risk of recurrent VTE and MB vs. warfarin.

## 1. Introduction

Venous thromboembolism (VTE) includes deep venous thrombosis (DVT) and pulmonary embolism (PE) and affects approximately 900,000 individuals annually in the United States [1,2]. Of these individuals, over 70,000 are severely obese patients, and these numbers are expected to increase [3,4]. Obesity is a known risk factor for new onset and recurrent VTE [5]. Current guidelines recommend patients with acute VTE initiate direct oral anticoagulants (DOACs) to help prevent recurrence [6]. However, anticoagulation therapy is complicated by certain safety concerns, primarily the risk of bleeding [7]. While the relationship between obesity and the risk of bleeding remains to be fully understood, obesity has been associated with an increased risk of major bleeding (MB) and clinically relevant nonmajor (CRNM) bleeding among patients treated with warfarin [8]. Anticoagulant therapy for obese patients is further complicated by the challenges to use fixed-dose treatments among VTE patients with extremely high body weights [9]. In 2016, the International Society on Thrombosis and Hemostasis (ISTH) recommended standard dosing of DOACs in obese patients with a body mass index (BMI) ≤ 40 or weight ≤ 120 kg for VTE treatment and prevention, but discouraged their use in morbidly obese patients with BMI > 40 or body weight > 120 kg due to inadequate clinical data [10,11].

Most phase-III DOAC VTE clinical studies have been characterized by relatively low sample sizes for obese patients [12,13,14,15]. These studies have reported no significant differences in efficacy between DOACs (apixaban, dabigatran, edoxaban, rivaroxaban) and warfarin, among obese patients with VTE [12,13,14,15]. A recent meta-analysis of anticoagulant use among obese patients identified two retrospective studies of obese patients with VTE prescribed apixaban vs. warfarin, with both showing apixaban non-inferior for recurrent VTE and non-inferior or superior for bleeding outcomes [16]. However, both studies were limited in sample size and generalizability. Therefore, the aim of this study was to use a large, nationally representative integrated dataset from five US health claims databases to compare the risk of recurrent VTE, MB, and CRNM bleeding among VTE patients with obesity and morbid obesity who initiated apixaban or warfarin.

## 2. Experimental Section

### 2.1. Data Source and Patient Selection

This study utilized pooled data from the Centers for Medicare & Medicaid Services (CMS) fee-for-service Medicare data and four US commercial claims databases: IBM^®^ MarketScan^®^ Commercial Claims and Encounter and Medicare Supplemental and Coordination of Benefits Database (MarketScan), IQVIA PharMetrics Plus™ (PharMetrics), Optum Clinformatics™ Data Mart (Optum), and the Humana^®^ Research Database (Humana).

**Figure 1:**
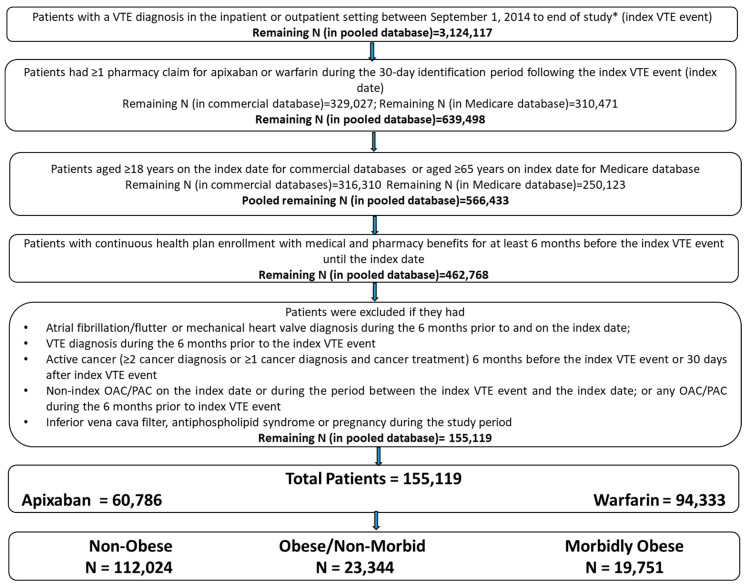
Patient selection criteria. * Study period: MarketScan: 1 March 2014–30 September 2018; Optum and Humana: 1 March 2014–31 December 2018; PharMetrics: 1 March 2014–31 March 2019; Medicare: 1 March 2014–31 December 2017. OAC: oral anticoagulant; PAC: parenteral anticoagulant; VTE: venous thromboembolism. Note: Medicare patients were restricted to those aged ≥65 years to avoid double counting of patients aged <65 years with dual Medicare/commercial coverage and to exclude younger patients eligible only due to end stage renal disease or disability.

Patients with ≥1 medical claim for VTE in any position (index VTE event) in the inpatient or outpatient setting were identified from 1 September 2014 until the end of available data. Patients aged ≥18 years (for commercial databases and ≥65 years for the Medicare database) were selected if they had ≥1 pharmacy claim for warfarin or apixaban during the 30-day period following the index VTE event. The first warfarin or apixaban prescription date was designated as the index date. Patients were required to have continuous health plan enrollment with medical and pharmacy benefits for 6 months prior to the index VTE event until the index date. Additional selection criteria are listed in Figure 1. The baseline period was defined as 6 months prior to and including the index date. Patients were followed from the day after the index date through the earliest of: the end of the subsequent 6-month period; index therapy discontinuation; switch to another OAC or parenteral anticoagulant (PAC) treatment; health plan disenrollment; death; or study end. The end of study date was different for each database, based on the data availability at the time of analysis (MarketScan: 01MAR2014-30SEP2018; Optum & Humana: 01MAR2014-31DEC2018; PharMetrics: 01MAR2014-31MAR2019; Medicare: 01MAR2014-31DEC2017).

### 2.2. Patient Cohorts

1. Obese Patients: Obesity was identified based on diagnosis codes that indicated obesity or codes that indicated a BMI of at least 30 (International Classification of Diseases, Ninth Clinical Modification [ICD-9-CM]: 278, V85.3, V85.4; International Classification of Diseases, Tenth Revisions, Clinical Modification [ICD-10-CM]: E66, Z68.3, Z68.4). Obese patients were further classified as morbidly obese and obese/non-morbid.
  - Morbidly obese patients were identified based on diagnosis codes that indicated morbid obesity or codes that indicated a BMI of at least 40 (ICD-9-CM: 278.01, V85.4; ICD-10-CM: E66.01, E66.2, Z68.4).
  - The remaining obese patients that were not classified as morbidly obese were designated as obese/non-morbid.
2. Non-Obese Patients: Patients not classified as obese were categorized as non-obese patients.

### 2.3. Outcome Measures

Clinical outcomes including recurrent VTE, MB, and CRNM bleeding were evaluated. Definitions of these clinical outcomes were based on previous publications [17,18,19,20]. Recurrent VTE was defined by primary/first-listed diagnosis in an inpatient setting, excluding admissions that occurred within 7 days of the index VTE encounter. ICD diagnosis codes were used to identify recurrent VTE events. MB events were also identified by primary/first-listed diagnosis in the inpatient setting and stratified by gastrointestinal (GI) bleeding, intracranial hemorrhage (ICH), and bleeding at other sites (genitourinary bleeding, respiratory tract bleeding, ocular bleeding, joint bleeding/hemarthrosis, transfusion of blood and blood components, other bleeding, or no bleeding site specified). CRNM bleeding was identified as either inpatient admission with a secondary diagnosis codes for non-critical sites of bleeding (excluded if MB occurred before the CRNM bleed or during the same hospitalization), or a diagnosis code for GI bleeding or other selected non-critical site of bleeding in the outpatient setting. Patients were censored upon recurrent VTE, MB, or CRNM bleeding events for the respective analysis and all the outcomes were measured independently.

Demographic (such as age and gender) and clinical characteristics (such as the Deyo-Charlson comorbidity index [CCI], baseline comorbidities, falls, fracture/trauma involving lower extremities, selected surgeries, and baseline medications) were evaluated during the baseline period. VTE-related variables, including the index VTE setting (inpatient vs. outpatient), the index VTE event type (DVT only vs. PE with or without DVT), and the index VTE etiology (unprovoked vs. provoked (defined as index VTE event that were preceded within 3 months by hormone therapy, fracture/trauma involving lower extremities, pelvic/orthopedic surgery, or hospitalization for any reason for ≥3 days)) [21] were measured on the index VTE event date.

### 2.4. Statistical Methods

Stabilized inverse probability treatment weighting (IPTW) was conducted within each database to balance patient characteristics between the treatment cohorts. Propensity scores were used to obtain estimates of the average treatment effect using a logistic model with the two treatment cohorts [22]. Covariates included in the propensity score model were demographics, clinical characteristics, and VTE-related variables (see Supplementary Tables S1 and S2 for details). After the propensity score calculation was made, each patient was weighted by the inverse of the probability of their treatment option (weight = 1/propensity score). The weights were stabilized by multiplying the original weights with a constant, which was equal to the expected value of being in the treatment or comparison cohorts, respectively [23,24,25]. After IPTW, the baseline characteristics were well balanced in each of the five databases, and patients were pooled for further analysis.

The risk of recurrent VTE, MB, and CRNM bleeding in each weighted cohort was evaluated using Cox proportional hazard models. Kaplan Meier curves were visually inspected to ensure the proportional hazard assumption was not violated. Interaction analysis was conducted to evaluate the treatment effect across the following 3 subgroups: non-obese, obese/non-morbid, morbidly obese. The statistical significance (*p* < 0.10) of the interaction between treatment and obesity status on effectiveness and safety were evaluated. Additionally, IPTW was conducted separately among patients with obesity and patients with morbid obesity to ensure baseline characteristics were well balanced between treatment cohorts for these subgroups; Cox proportional hazard models were used to evaluate the risk of clinical outcomes. No covariates were included in the models as they were balanced.

## 3. Results

After applying the selection criteria, a total of 155,119 VTE patients including 60,786 (39.2%) who initiated apixaban and 94,333 (60.8%) who initiated warfarin were identified in the pooled database (Figure 1). Of the total population, 112,024 (72.2%) were categorized as non-obese, 23,344 (15.0%) as obese/non-morbid, and 19,751 (12.7%) as morbidly obese VTE patients.

Table 1 shows the baseline characteristics of non-obese, obese/non-morbid, and morbidly obese patients receiving apixaban or warfarin before IPTW: morbidly obese (mean age: 62 years; CCI: 2.9) and obese/non-morbid (mean age: 66 years; CCI: 2.5) patients were younger and had a higher baseline CCI compared to non-obese (mean age: 68 years; CCI: 2.0) patients. Morbidly obese and obese/non-morbid patients were more likely to be diagnosed with PE (57.2% and 48.2% vs. 40.1%) and higher proportions experienced provoked VTE events (65.7% and 61.8% vs. 53.4%) compared to non-obese patients. Morbidly obese and obese/non-morbid patients were also more likely to have comorbidities such as hypertension (82.9% and 77.7% vs. 64.5%), hyperlipidemia (55.5% and 58.7% vs. 44.6%), and diabetes (48.7% and 38.2% vs. 25.7%) compared to non-obese patients.

**Figure 2:**
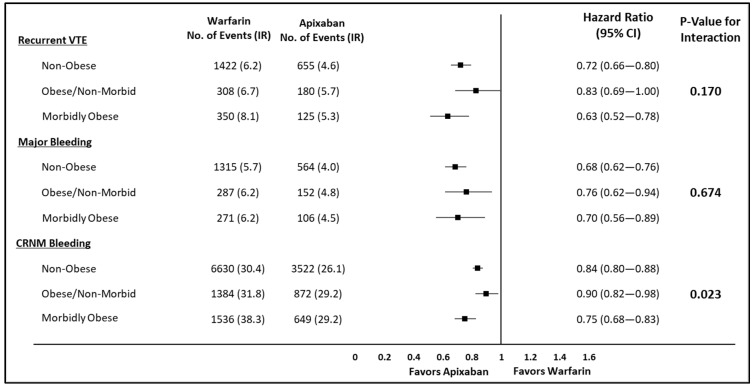
Risk of recurrent VTE, major bleeding, and CRNM bleeding among VTE patients stratified by obesity status. CI: confidence interval; CRNM: clinically relevant non-major; IR: incidence rate (per 100 person-years); VTE: venous thromboembolism. CI: Confidence Interval; CRNM: Clinically Relevant non-major; IR: Incidence Rate; VTE: Venous Thromboembolism.

After applying IPTW for the apixaban and warfarin cohorts, baseline patient characteristics were balanced between the two cohorts (Supplemental Table S1). Figure 2 represents the hazard ratios for the comparison of recurrent VTE, MB, and CRNM bleeding between apixaban and warfarin in the IPTW weighted population stratified by non-obese vs. obese/non-morbid vs. morbidly obese. No significant interaction was observed between treatment and obesity status for recurrent VTE (interaction *p* = 0.170) and MB (interaction *p* = 0.674). One significant interaction was observed for CRNM bleeding (interaction *p* = 0.023): while apixaban trended towards lower risk of CRNM bleeding compared to warfarin across non-obese, obese/non-morbid, and morbidly obese patients; the magnitude of the difference was bigger for morbidly obese patients vs. the other two subgroups.

The pre- and post-IPTW baseline characteristics among VTE patients who were obese or morbidly obese are listed in the Supplemental Tables S2 and S3, respectively. After applying IPTW to the obese patients, baseline patient characteristics were balanced between the apixaban and warfarin cohorts; the mean age was 64 years with a mean CCI score of 2.7, approximately 64% patients had provoked VTE events, and 23% of patients had a history of bleeding at baseline. In the post-IPTW morbidly obese population, the baseline patient characteristics were balanced between the two treatment cohorts and the mean age was 62 years with a mean CCI score of 2.9.

**Figure 3:**
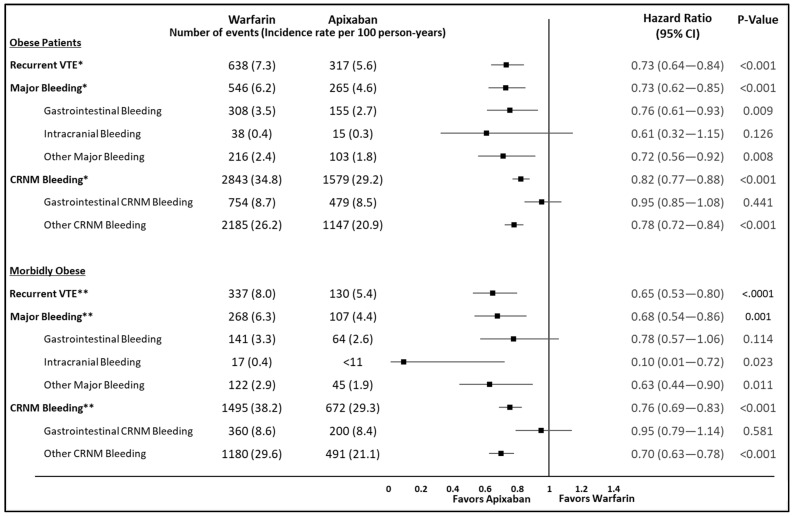
Risk of recurrent VTE, major bleeding, and CRNM bleeding among IPTW weighted obese and morbidly obese VTE patients prescribed apixaban vs. warfarin. CI: confidence interval; CRNM: clinically relevant non-major; IPTW: inverse probability treatment weighting; IR: incidence rate (per 100 person-years); VTE: venous thromboembolism; CMS: Centers for Medicare & Medicaid Services. Note: CMS data user agreements preclude reporting of results with *n* < 11. CI: Confidence Interval; CRNM: Clinically Relevant non-major; IR: Incidence Rate; VTE: Venous Thromboembolism. * Incidence rate for recurrent VTE (per 100 person years [95%confidence interval]): Warfarin (7.3 [6.8,7.9]); Apixaban (5.6 [5.0,6.2]); Total person -years for recurrent VTE: Warfarin (8,735.4); Apixaban (5,688.5)**;** Incidence rate for Major Bleeding (per 100 person years [95%confidence interval]): Warfarin (6.2 [5.73,6.8]); Apixaban (4.6 [4.1,5.2]); Total person -years for Major Bleeding: Warfarin (8,762.4); Apixaban (5,706.6)**;** Incidence rate for CRNM Bleeding (per 100 person years [95%confidence interval]): Warfarin (34.8 [33.5,36.1]); Apixaban (29.2 [27.8,30.7]); Total person -years for CRNM Bleeding: Warfarin (8,181.4); Apixaban (5,397.7); ** Incidence rate for recurrent VTE (per 100 person years [95%confidence interval]): Warfarin (8.0 [7.2,8.9]); Apixaban (5.4 [4.5,6.4]); Total person -years for recurrent VTE: Warfarin (4,212.2); Apixaban (2,412.9)**;** Incidence rate for Major Bleeding (per 100 person years [95%confidence interval]): Warfarin (6.3 [5.6,7.2]); Apixaban (4.4 [3.6,5.33]); Total person -years for Major Bleeding: Warfarin (4,230.8); Apixaban (2,418.8)**;** Incidence rate for CRNM Bleeding (per 100 person years [95%confidence interval]): Warfarin (38.2 [36.3,40.1]); Apixaban (29.3 [27.2,31.6]); Total person -years for CRNM Bleeding: Warfarin (3,919.5); Apixaban (2,290.4).

Supplemental Table S4 shows the incidence rate of recurrent VTE, major bleeding, and CRNM bleeding among obese and morbidly obese VTE patients that initiated apixaban vs. warfarin. Among obese patients, apixaban was associated with a significantly lower risk of recurrent VTE (HR: 0.73; 95% CI: 0.64–0.84), MB (HR: 0.73; 95% CI: 0.62–0.85), and CRNM bleeding (HR: 0.82; 95% CI: 0.77–0.88) compared to warfarin (Figure 3). Similarly among morbidly obese patients, apixaban was associated with a significantly lower risk of recurrent VTE (HR: 0.65; 95% CI: 0.53–0.80), MB (HR: 0.68; 95% CI: 0.54–0.86), and CRNM bleeding (HR: 0.76; 95% CI: 0.69–0.83) compared to warfarin (Figure 3).

## 4. Discussion

This real-world study integrated patient information from Medicare and 4 major national commercial databases to construct a large and highly representative sample of VTE patients initiating apixaban or warfarin in the United States. Of these patients, those who were obese/non-morbid and morbidly obese had notably different patient characteristics as compared to non-obese patients, especially regarding baseline comorbidity. However, the effects of apixaban vs. warfarin on recurrent VTE and MB were consistent regardless of the obesity status. Further analyses of obese and morbidly obese patients respectively showed that patients who initiated apixaban had a significantly lower risk of recurrent VTE, MB, and CRNM bleeding as compared to warfarin patients.

Recent ISTH guidelines for the treatment of VTE recommend avoiding DOAC use in patients who weigh > 120 kg due to limited clinical data in this population [11]. In the AMPLIFY trial, apixaban was noninferior to enoxaparin followed by warfarin for the treatment of VTE, with a significantly lower risk of MB [12]. Generally consistent findings were observed across the weight and BMI subgroups, as no significant interaction was reported between treatment and weight for recurrent VTE (*p* = 0.4344) or MB (*p* = 0.3210) or between treatment and the BMI for MB (*p* = 0.4773) [12]. A post hoc analysis of the AMPLIFY clinical trial showed that efficacy and safety of apixaban in patients with extreme body weight (≥120 kg) were consistent with the main results from the AMPLIFY trial [26].

The results of the current study were generally consistent with the AMPLIFY trial and post-hoc analysis, wherein no significant interaction was observed between treatment of apixaban vs. warfarin and obesity status for recurrent VTE and MB. Beyond the interaction analyses, the current study further analyzed the effectiveness and safety of apixaban vs. warfarin among VTE patients with obesity and morbid obesity, respectively. Among the subgroups of obese and morbidly obese patients, those treated with apixaban had a lower risk of recurrent VTE and MB as compared with those treated with warfarin. The current study, which reflects real world practice and challenges (for example, non-compliance), provides complementary information to the AMPLIFY trial, further supporting the benefits of apixaban vs. warfarin in VTE patients with obesity as well as morbid obesity.

There is currently very limited evidence in the literature regarding the use of DOACs in obese and morbidly obese VTE patients. A Mayo Clinic VTE registry study reported that the rate of recurrent VTE was numerically lower in patients using DOACs (apixaban or rivaroxaban) compared to the monitored anticoagulation therapy (low-molecular-weight heparin [LMWH] or warfarin) for body weights < 60 kg (1.49 vs. 5.31 per 100 person-years), 60–120 kg (3.19 vs. 4.85 per 100 person-years), and >120 kg (4.91 vs. 10.55 per 100 person-years) [27]. Similarly, the incidence of MB was numerically lower for DOACs vs. LMWH or warfarin for body weights < 60 kg (6.06 vs. 10.89 per 100 person-years) and >120 kg (2.40 vs. 8.45 per 100 person-years). For 60–120 kg, DOACs had a significantly lower rate of MB (2.69 vs. 5.64 per 100 person-years) as compared with LMWH/warfarin. A couple of retrospective studies with smaller sample sizes than the current study compared effectiveness and safety outcomes associated with apixaban and warfarin among morbidly obese patients. These studies found apixaban to be noninferior for recurrent VTE and noninferior or superior for MB and CRNM bleeding as compared to warfarin [28,29]. Another retrospective single-center study reported that no differences were observed in recurrent VTE and bleeding events between DOACs and warfarin [30]. However, about 92% of the patients had initiated rivaroxaban, 3% of patients initiated dabigatran, and only 5% patients initiated apixaban, rendering the results not directly comparable to the current study. With a much bigger sample size and pooling of patients from 5 national claims databases, the current study aids in addressing some of the data gaps regarding DOAC, more specifically apixaban use in VTE patients with obesity and morbid obesity.

### Limitations

This study has some inherent limitations. Firstly, only association, not causality, can be inferred from this study due to its observational design. Moreover, the study employed an on-treatment approach that did not evaluate events occurring after patients switched or discontinued the index treatment. While some of these events may be related to the index drug, it is difficult to confirm the relationship based on claims data. This study also employed the IPTW weighting system to control for varying patient characteristics while preserving all eligible study subjects, but the IPTW methodology may be subject to some hidden or residual confounding (for example, confounding by indication) and should be interpreted accordingly. The definitions of recurrent VTE and MB were based on inpatient claims with primary or first-listed diagnosis codes for VTE or MB. Although the codes have previously been validated in the literature, the presence/absence of diagnosis codes does not always equate with a presence/absence of disease. VTE events occurring in the outpatient setting were not included in the evaluation of recurrent VTE as it is difficult to separate true recurrent events from follow-up consultations in the outpatient setting and the positive predictive value for outpatient recurrent VTE is usually low. [31] Hence, certain clinical outcomes might be under or overestimated. Due to the lack of weight information in the databases, obesity was identified based on ICD diagnosis codes for BMI ≥ 30 or codes that indicated obesity. Similarly, morbid obesity was identified based on ICD diagnosis codes for BMI ≥ 40 or codes that had an indication for morbid obesity. However, a previous study in the atrial fibrillation population had validated the ICD diagnosis codes for obesity and morbid obesity using one of the databases used in the current study linked with electronic medical records. The study reported a high positive predictive value for obesity and modest for morbidly obese (PPV; 89.8% and 67.9%), high specificity (95.2% and 96.5%), and modest sensitivity (48.7% and 63.8%) for obesity and morbidly obese diagnosis codes, respectively, among newly treated nonvalvular atrial fibrillation patients. [32] Therefore, while it is possible that a number of obese patients were missed, the large majority of patients who were defined as obese are very likely to be obese by clinical measures. Another limitation is that there is a possibility that patients could be insured by more than one health plan and, as such, duplicate patients could not be excluded from the pooled database. However, prior literature has reported only 0.5% duplicates between two databases [33] and hence, this should not impact the study results. Furthermore, death data was not collected across all the data sources used in this study. For a couple of data sources that did collect death information, observable death rates were too low to expect any big impacts on the study results. However, the absence of complete death data precluded evaluation of death as a competing risk in risk analyses, and results should be interpreted accordingly. Finally, the results may not be generalizable to the entire obese VTE population in the United States as we did not include patients that were uninsured or those who had coverage through other insurance (for example, private insurance, Veterans Affairs, Medicaid).

## 5. Conclusions

This study evaluated the effectiveness and safety outcomes associated with two anticoagulation therapies among obese and morbidly obese VTE patients, using a large, integrated national dataset. Results showed that among both obese and morbidly obese patients, the use of apixaban was associated with significantly lower risk of recurrent VTE, MB, and CRNM bleeding as compared with warfarin therapy. These results add much needed evidence to the limited data about the use of DOACs in obese VTE patients and call for continued research that may better inform clinical decision-making for this vulnerable and growing patient population.

## Figures and Tables

**Table 1:** Descriptive baseline and demographic characteristics among non-obese, obese/non-morbid, and morbidly obese patients.

	Non-Obese	Obese/Non-Morbid	STD *	Morbidly Obese	STD *
Sample Size	112,024	23,344		19,751	
Age, Mean (SD)	67.5 (16.5)	66.2 (14.2)	8.77	62.1 (14.0)	35.31
Age Categories, *n* (%)					
18–54	23,553 (21.0%)	4737 (20.3%)	1.81	5510 (27.9%)	16.04
55–64	17,641 (15.7%)	3915 (16.8%)	2.77	3937 (19.9%)	10.95
65–74	29,065 (25.9%)	7890 (33.8%)	17.22	6870 (34.8%)	19.31
75–79	13,840 (12.4%)	3129 (13.4%)	3.13	1948 (9.9%)	7.94
≥80	27,925 (24.9%)	3673 (15.7%)	22.99	1486 (7.5%)	48.58
Gender, *n* (%)					
Male	52,410 (46.8%)	10,482 (44.9%)	3.78	7219 (36.6%)	20.87
Female	59,614 (53.2%)	12,862 (55.1%)	3.78	12,532 (63.4%)	20.87
Index VTE Setting, *n* (%)					
Inpatient	56,065 (50.0%)	14,103 (60.4%)	20.96	13,654 (69.1%)	39.64
Outpatient	55,959 (50.0%)	9241 (39.6%)	20.96	6097 (30.9%)	39.64
Index VTE Event Type, *n* (%)					
DVT Only	67,055 (59.9%)	12,103 (51.8%)	16.19	8458 (42.8%)	34.59
PE with or without DVT	44,969 (40.1%)	11,241 (48.2%)	16.19	11,293 (57.2%)	34.59
Index VTE Etiology, *n* (%)					
Provoked	59,790 (53.4%)	14,437 (61.8%)	17.21	12,978 (65.7%)	25.33
Unprovoked	52,234 (46.6%)	8907 (38.2%)	17.21	6773 (34.3%)	25.33
Deyo-Charlson Comorbidity Index, Mean (SD)	2.0 (2.3)	2.5 (2.5)	21.89	2.9 (2.6)	36.28
Baseline Comorbidity, *n* (%)					
Anemia	31,341 (28.0%)	7616 (32.6%)	10.13	6506 (32.9%)	10.80
Central Venous Catheter	7925 (7.1%)	2144 (9.2%)	7.73	2493 (12.6%)	18.70
** Hematologic disorders associated with bleeding	8612 (7.7%)	2099 (9.0%)	4.72	1710 (8.7%)	3.54
Ischemic Heart/ Coronary Artery Disease	26,614 (23.8%)	6931 (29.7%)	13.44	5705 (28.9%)	11.66
Dementia	8584 (7.7%)	965 (4.1%)	15.02	534 (2.7%)	22.51
Dyspepsia or Stomach Discomfort	24,365 (21.7%)	6049 (25.9%)	9.78	4932 (25.0%)	7.62
Hyperlipidemia	49,908 (44.6%)	13,695 (58.7%)	28.53	10,955 (55.5%)	21.96
Sleep Apnea	8597 (7.7%)	4717 (20.2%)	36.79	7476 (37.9%)	77.14
*** Thrombophilia	4214 (3.8%)	1028 (4.4%)	3.24	848 (4.3%)	2.70
Varicose Veins	4357 (3.9%)	1082 (4.6%)	3.69	1132 (5.7%)	8.62
Congestive Heart Failure	16,465 (14.7%)	4384 (18.8%)	10.95	5055 (25.6%)	27.42
Diabetes	28,796 (25.7%)	8921 (38.2%)	27.07	9614 (48.7%)	48.93
Hypertension	72,297 (64.5%)	18,148 (77.7%)	29.46	16,364 (82.9%)	42.53
Non-ESRD Renal Disease	14,803 (13.2%)	4199 (18.0%)	13.18	3846 (19.5%)	16.99
End Stage Renal Disease	2442 (2.2%)	587 (2.5%)	2.21	541 (2.7%)	3.61
Chronic Liver Disease	6917 (6.2%)	2117 (9.1%)	10.92	1981 (10.0%)	14.16
Chronic Obstructive Pulmonary Disease	19,315 (17.2%)	4560 (19.5%)	5.92	4577 (23.2%)	14.81
Baseline Bleed	22,089 (19.7%)	5418 (23.2%)	8.51	4421 (22.4%)	6.54

DVT: deep venous thrombosis; ESRD: end stage renal disease; PE: pulmonary embolism; SD: standard deviation; STD: standardized differences; VTE: venous thromboembolism. * STD vs. non-obese. STD = 100 *|actual STD|. STD > 10.00 is considered significant. ** Hematologic disorders associated with bleeding: conditions that hinder mediation of blood clotting and increase bleeding risk, e.g., Von Willebrand’s disease, the defibrination syndrome, acquired coagulation factor deficiency, unspecified coagulation defects, allergic purpura, qualitative platelet defects, nonthrombocytopenic purpuras, thrombocytopenia, and thrombotic microangiopathy. *** Thrombophilia: conditions that increase the risk of blood clot development, e.g., diseases of blood and blood-forming organs, thalassemia, polycythemia vera, prothrombin gene mutation, and lupus anticoagulant syndrome.

## Data Availability

The data presented in the study are not publicly available due to the data user agreement between authors and CMS Medicare.

## Supplementary Materials

The following are available online at https://www.mdpi.com/2077-0383/10/2/200/s1, Table S1: Post IPTW Descriptive Baseline Characteristics among Overall VTE Patients who Initiated Apixaban vs Warfarin, Table S2: Pre- and Post-IPTW Descriptive Baseline Characteristics Among VTE Patients with Obesity who Initiated Apixaban vs Warfarin, Table S3: Pre and post IPTW Descriptive Baseline Characteristics among VTE patients with morbid obesity who initiated apixaban vs warfarin, Table S4: Post IPTW Incidence Rate of Recurrent VTE, Major Bleeding, and CRNM Bleeding among Obese and Morbid Obese VTE Patients who initiated Apixaban vs. Warfarin.
