# Supplementary material for: Effectiveness and Safety of Apixaban vs. Warfarin in Venous Thromboembolism Patients with Obesity and Morbid Obesity
Source: J Clin Med. 2021 Jan 8;10(2):200. doi: 10.3390/jcm10020200 (PMC7827069; doi:10.3390/jcm10020200)
Supplement: Supplementary file 1 [file jcm-10-00200-s001.pdf]

Supplemental Table 1. Post IPTW Descriptive Baseline Characteristics among Overall VTE Patients who Initiated Apixaban vs Warfarin

		Post-IPTW Warfarin Cohort (Reference)	Apixaban Cohort	STD[a]
Sample Size		94,333	60,786	
Age, Mean (SD)		66.7 (15.9)	66.8 (16.0)	0.80
Age Categories,[b] n(%)				
	18-54	20,449 (21.7%)	13,098 (21.5%)	0.31
	55-64	15,430 (16.4%)	9,908 (16.3%)	0.15
	65-74	26,709 (28.3%)	17,219 (28.3%)	0.03
	75-79	11,545 (12.2%)	7,471 (12.3%)	0.16
	≥80	20,200 (21.4%)	13,089 (21.5%)	0.29
Gender, n(%)				
	Male	42,590 (45.1%)	27,410 (45.1%)	0.11
	Female	51,743 (54.9%)	33,376 (54.9%)	0.11
Geographic Region,[c] n(%)				
	Northeast	14,825 (15.7%)	9,494 (15.6%)	0.27
	North Central	26,271 (27.8%)	16,873 (27.8%)	0.20
	South	35,815 (38.0%)	23,008 (37.9%)	0.24
	West	17,257 (18.3%)	11,301 (18.6%)	0.77
	Other	165 (0.2%)	110 (0.2%)	0.16
Index VTE Setting, n(%)				
	Inpatient	51,097 (54.2%)	33,030 (54.3%)	0.35
	Outpatient	43,236 (45.8%)	27,756 (45.7%)	0.35
Index VTE Event Type, n(%)				
	DVT only	53,279 (56.5%)	34,367 (56.5%)	0.12
	PE with or without DVT	41,054 (43.5%)	26,419 (43.5%)	0.12
Index VTE Etiology, n(%)				
	Provoked	53,076 (56.3%)	34,264 (56.4%)	0.21
	Unprovoked	41,257 (43.7%)	26,522 (43.6%)	0.21
Deyo-Charlson Comorbidity Index, Mean (SD)		2.2 (2.4)	2.2 (2.4)	0.71
Baseline Comorbidity, n(%)				
	Alcohol abuse	2,987 (3.2%)	1,937 (3.2%)	0.12
	Anemia	27,751 (29.4%)	17,985 (29.6%)	0.37
	Central venous Catheter	7,672 (8.1%)	4,977 (8.2%)	0.20
	Hematologic disorders associated with bleeding	7,611 (8.1%)	5,000 (8.2%)	0.58
	Ischemic heart/ coronary artery disease	23,951 (25.4%)	15,491 (25.5%)	0.22

Dyspepsia or stomach discomfort	21,551 (22.8%)	13,960 (23.0%)	0.28
Hyperlipidemia	45,444 (48.2%)	29,333 (48.3%)	0.16
Obesity	26,218 (27.8%)	16,896 (27.8%)	0.01
Pneumonia	13,641 (14.5%)	8,819 (14.5%)	0.13
Rheumatologic disease	4,430 (4.7%)	2,854 (4.7%)	0.01
Sleep apnea	12,634 (13.4%)	8,118 (13.4%)	0.11
Spinal cord injury	253 (0.3%)	168 (0.3%)	0.15
Thrombophilia	3,718 (3.9%)	2,420 (4.0%)	0.21
Varicose Veins	3,988 (4.2%)	2,560 (4.2%)	0.08
Hypertension	65,067 (69.0%)	42,014 (69.1%)	0.31
Non-ESRD Renal Disease	12,837 (13.6%)	8,297 (13.6%)	0.12
End Stage Renal Disease	2,179 (2.3%)	1,434 (2.4%)	0.33
Inflammatory Bowel Disease	1,769 (1.9%)	1,132 (1.9%)	0.10
Baseline bleed	19,481 (20.7%)	12,611 (20.7%)	0.24
Recent History of Falls, n(%)	6,853 (7.3%)	4,426 (7.3%)	0.06
Fracture/trauma involving Lower Extremities, n(%)	14,515 (15.4%)	9,346 (15.4%)	0.03
Selected Surgeries, n(%)	23,353 (24.8%)	15,109 (24.9%)	0.23
Baseline Medication Use, n(%)			
Antiarrhythmic	9,044 (9.6%)	5,841 (9.6%)	0.07
Statins	36,293 (38.5%)	23,437 (38.6%)	0.17
Anti-platelets	7,140 (7.6%)	4,613 (7.6%)	0.08
Aromatase Inhibitors	207 (0.2%)	131 (0.2%)	0.06
Beta Blockers	32,108 (34.0%)	20,784 (34.2%)	0.33
Gastroprotective Agents	26,741 (28.3%)	17,274 (28.4%)	0.16
SERMS	567 (0.6%)	364 (0.6%)	0.04
NSAIDs	24,770 (26.3%)	15,951 (26.2%)	0.04
Hormone Therapy (estrogen)	5,207 (5.5%)	3,344 (5.5%)	0.08

DVT: Deep vein thrombosis; ESRD: End stage renal disease; IPTW: Inverse probability treatment weighting; NSAIDs: Nonsteroidal anti-inflammatory drug; SERMS: Selective estrogen receptor modulator; PE: Pulmonary embolism; SD: Standard deviation; STD: Standardized differences; VTE: Venous thromboembolism

[a] Std Difference=100*|actual std diff|. Std Difference greater than 10 is considered significant

[b] After applying weights, the values for age category were not whole numbers; therefore, due to rounding the sum of patients does not equal 100%

[c] After applying weights, the values for region category were not whole numbers; therefore, due to rounding the sum of patients does not equal 100%

Note: the following variables were considered in the propensity score model and subsequent weighting: age category, gender, geographic region, type of index encounter (inpatient vs outpatient), VTE diagnosis (DVT vs PE with or without DVT), VTE etiology (provoked vs unprovoked), Deyo Charlson comorbidity

index, baseline comorbidities (all listed tabs), recent history of falls, fracture/trauma involving lower extremities, selected surgeries, and baseline medication use (weighted for specific medications).

Supplemental Table 2. Pre- and Post-IPTW Descriptive Baseline Characteristics Among VTE Patients with Obesity who Initiated Apixaban vs Warfarin

	Pre-IPTW			Post-IPTW		
	**Warfarin Cohort (Reference)**	**Apixaban Cohort**	**STD[a]**	**Warfarin Cohort (Reference)**	**Apixaban Cohort**	**STD[a]**
Sample Size	**25,602**	**17,493**		**25,602**	**17,493**	
Age, Mean (SD)	64.9 (14.1)	63.5 (14.5)	9.61	64.3 (14.3)	64.5 (14.2)	1.41
Age Categories,[b] n(%)						
18-54	5,702 (22.3%)	4,545 (26.0%)	8.68	6,074 (23.7%)	4,130 (23.6%)	0.27
55-64	4,276 (16.7%)	3,576 (20.4%)	9.63	4,635 (18.1%)	3,155 (18.0%)	0.18
65-74	9,358 (36.6%)	5,402 (30.9%)	12.02	8,781 (34.3%)	6,008 (34.3%)	0.10
75-79	3,120 (12.2%)	1,957 (11.2%)	3.11	3,034 (11.9%)	2,089 (11.9%)	0.27
≥80	3,146 (12.3%)	2,013 (11.5%)	2.41	3,078 (12.0%)	2,112 (12.1%)	0.15
Gender, n(%)						
Male	10,120 (39.5%)	7,581 (43.3%)	7.74	10,504 (41.0%)	7,167 (41.0%)	0.12
Female	15,482 (60.5%)	9,912 (56.7%)	7.74	15,098 (59.0%)	10,326 (59.0%)	0.12
Geographic Region,[c] n(%)						
Northeast	4,059 (15.9%)	2,463 (14.1%)	4.97	3,869 (15.1%)	2,626 (15.0%)	0.28
North Central	8,088 (31.6%)	3,845 (22.0%)	21.83	7,094 (27.7%)	4,831 (27.6%)	0.20
South	8,829 (34.5%)	9,162 (52.4%)	36.69	10,667 (41.7%)	7,275 (41.6%)	0.15
West	4,584 (17.9%)	2,011 (11.5%)	18.17	3,940 (15.4%)	2,737 (15.6%)	0.71
Other	42 (0.2%)	12 (0.1%)	2.80	32 (0.1%)	23 (0.1%)	0.19
Index VTE Setting, n(%)						
Inpatient	17,155 (67.0%)	10,602 (60.6%)	13.35	16,530 (64.6%)	11,321 (64.7%)	0.32
Outpatient	8,447 (33.0%)	6,891 (39.4%)	13.35	9,072 (35.4%)	6,172 (35.3%)	0.32
Index VTE Event Type, n(%)						
DVT only	12,183 (47.6%)	8,378 (47.9%)	0.62	12,214 (47.7%)	8,359 (47.8%)	0.16
PE with or without DVT	13,419 (52.4%)	9,115 (52.1%)	0.62	13,388 (52.3%)	9,134 (52.2%)	0.16
Index VTE Etiology, n(%)						
Provoked	17,000 (66.4%)	10,415 (59.5%)	14.25	16,298 (63.7%)	11,146 (63.7%)	0.13
Unprovoked	8,602 (33.6%)	7,078 (40.5%)	14.25	9,304 (36.3%)	6,347 (36.3%)	0.13

Deyo-Charlson Comorbidity Index, Mean (SD)	2.8 (2.6)	2.5 (2.5)	10.11	2.7 (2.6)	2.7 (2.6)	0.53
Baseline Comorbidity, n(%)						
Alcohol abuse	684 (2.7%)	456 (2.6%)	0.40	684 (2.7%)	475 (2.7%)	0.26
Anemia	8,958 (35.0%)	5,164 (29.5%)	11.72	8,405 (32.8%)	5,765 (33.0%)	0.27
Central venous Catheter	3,033 (11.8%)	1,604 (9.2%)	8.74	2,772 (10.8%)	1,909 (10.9%)	0.28
Hematologic disorders associated with bleeding	2,521 (9.8%)	1,288 (7.4%)	8.87	2,270 (8.9%)	1,564 (8.9%)	0.25
Ischemic heart/ coronary artery disease	7,599 (29.7%)	5,037 (28.8%)	1.95	7,551 (29.5%)	5,189 (29.7%)	0.37
Dyspepsia or stomach discomfort	6,608 (25.8%)	4,373 (25.0%)	1.87	6,549 (25.6%)	4,508 (25.8%)	0.43
Hyperlipidemia	14,732 (57.5%)	9,918 (56.7%)	1.71	14,683 (57.3%)	10,048 (57.4%)	0.19
Pneumonia	3,955 (15.4%)	2,639 (15.1%)	1.01	3,930 (15.4%)	2,681 (15.3%)	0.07
Rheumatologic disease	1,332 (5.2%)	845 (4.8%)	1.71	1,297 (5.1%)	891 (5.1%)	0.14
Sleep apnea	7,448 (29.1%)	4,745 (27.1%)	4.38	7,239 (28.3%)	4,932 (28.2%)	0.18
Spinal cord injury	67 (0.3%)	47 (0.3%)	0.14	67 (0.3%)	44 (0.3%)	0.21
Thrombophilia	1,180 (4.6%)	696 (4.0%)	3.11	1,125 (4.4%)	784 (4.5%)	0.42
Varicose Veins	1,267 (4.9%)	947 (5.4%)	2.10	1,310 (5.1%)	896 (5.1%)	0.03
Hypertension	20,774 (81.1%)	13,738 (78.5%)	6.50	20,529 (80.2%)	14,042 (80.3%)	0.22
Non-ESRD Renal Disease	4,965 (19.4%)	3,080 (17.6%)	4.60	4,810 (18.8%)	3,309 (18.9%)	0.33
End Stage Renal Disease	835 (3.3%)	293 (1.7%)	10.24	673 (2.6%)	469 (2.7%)	0.33
Inflammatory Bowel Disease	447 (1.7%)	280 (1.6%)	1.13	433 (1.7%)	291 (1.7%)	0.21
Baseline bleed	6,395 (25.0%)	3,444 (19.7%)	12.73	5,858 (22.9%)	4,012 (22.9%)	0.13
Recent History of Falls, n(%)	1,670 (6.5%)	1,077 (6.2%)	1.50	1,645 (6.4%)	1,127 (6.4%)	0.09
Fracture/trauma involving Lower Extremities, n(%)	3,798 (14.8%)	2,682 (15.3%)	1.39	3,844 (15.0%)	2,619 (15.0%)	0.12
Selected Surgeries, n(%)	8,225 (32.1%)	5,193 (29.7%)	5.28	8,015 (31.3%)	5,491 (31.4%)	0.18
Baseline Medication Use, n(%)						
Antiarrhythmic	2,955 (11.5%)	1,905 (10.9%)	2.07	2,881 (11.3%)	1,964 (11.2%)	0.09
Statins	11,332 (44.3%)	7,562 (43.2%)	2.08	11,268 (44.0%)	7,728 (44.2%)	0.33
Anti-platelets	2,084 (8.1%)	1,452 (8.3%)	0.58	2,110 (8.2%)	1,441 (8.2%)	0.02
Aromatase Inhibitors	51 (0.2%)	36 (0.2%)	0.15	52 (0.2%)	34 (0.2%)	0.19
Beta Blockers	10,283 (40.2%)	6,555 (37.5%)	5.53	10,026 (39.2%)	6,874 (39.3%)	0.27
Gastroprotective Agents	8,402 (32.8%)	5,514 (31.5%)	2.78	8,278 (32.3%)	5,666 (32.4%)	0.12
SERMS	99 (0.4%)	83 (0.5%)	1.34	110 (0.4%)	75 (0.4%)	0.02
NSAIDs	8,035 (31.4%)	5,990 (34.2%)	6.09	8,311 (32.5%)	5,671 (32.4%)	0.09
Hormone Therapy (estrogen)	1,225 (4.8%)	1,045 (6.0%)	5.27	1,331 (5.2%)	903 (5.2%)	0.15

DVT: Deep vein thrombosis; ESRD: End stage renal disease; IPTW: Inverse probability treatment weighting; NSAIDs: Nonsteroidal anti-inflammatory drug; SERMS: Selective estrogen receptor modulator; PE: Pulmonary embolism; SD: Standard deviation; STD: Standardized differences; VTE: Venous thromboembolism

[a] Std Difference=100*|actual std diff|. Std Difference greater than 10 is considered significant

[b] After applying weights, the values for age category were not whole numbers; therefore, due to rounding the sum of patients does not equal 100%

[c] After applying weights, the values for region category were not whole numbers; therefore, due to rounding the sum of patients does not equal 100%

Note: the following variables were considered in the propensity score model and subsequent weighting: age category, gender, geographic region, type of index encounter (inpatient vs outpatient), VTE diagnosis (DVT vs PE with or without DVT), VTE etiology (provoked vs unprovoked), Deyo Charlson comorbidity index, baseline comorbidities (all listed tabs), recent history of falls, fracture/trauma involving lower extremities, selected surgeries, and baseline medication use (weighted for specific medications).

Supplemental Table 3. Pre and post IPTW Descriptive Baseline Characteristics among VTE patients with morbid obesity who initiated apixaban vs warfarin

		**Pre-IPTW**			**Post-IPTW**	
	**Warfarin Cohort (Reference)**	**Apixaban Cohort**	**STD[a]**	**Warfarin Cohort (Reference)**	**Apixaban Cohort**	**STD[a]**
Sample Size	**12,340**	**7,411**		**12,340**	**7,411**	
Age, Mean (SD)	62.4 (13.9)	61.7 (14.0)	4.60	62.1 (14.0)	62.3 (13.9)	1.81
Age Categories,[b] n(%)						
18-54	3,356 (27.2%)	2,154 (29.1%)	4.16	3,438 (27.9%)	2,051 (27.7%)	0.41
55-64	2,325 (18.8%)	1,612 (21.8%)	7.24	2,453 (19.9%)	1,467 (19.8%)	0.22
65-74	4,481 (36.3%)	2,389 (32.2%)	8.60	4,297 (34.8%)	2,590 (35.0%)	0.28
75-79	1,250 (10.1%)	698 (9.4%)	2.40	1,221 (9.9%)	738 (10.0%)	0.21
≥80	928 (7.5%)	558 (7.5%)	0.03	932 (7.6%)	565 (7.6%)	0.28
Gender, n(%)						
Male	4,408 (35.7%)	2,811 (37.9%)	4.58	4,511 (36.6%)	2,702 (36.5%)	0.19
Female	7,932 (64.3%)	4,600 (62.1%)	4.58	7,829 (63.4%)	4,709 (63.5%)	0.19
Geographic Region,[c] n(%)						
Northeast	1,835 (14.9%)	988 (13.3%)	4.42	1,761 (14.3%)	1,050 (14.2%)	0.28
North Central	4,050 (32.8%)	1,661 (22.4%)	23.44	3,568 (28.9%)	2,139 (28.9%)	0.13
South	4,226 (34.2%)	3,931 (53.0%)	38.60	5,095 (41.3%)	3,058 (41.3%)	0.06
West	2,210 (17.9%)	823 (11.1%)	19.41	1,899 (15.4%)	1,154 (15.6%)	0.51
Other	19 (0.2%)	8 (0.1%)	1.27	17 (0.1%)	10 (0.1%)	0.05
Index VTE Setting, n(%)						
Inpatient	8,691 (70.4%)	4,963 (67.0%)	7.47	8,544 (69.2%)	5,146 (69.4%)	0.44
Outpatient	3,649 (29.6%)	2,448 (33.0%)	7.47	3,796 (30.8%)	2,265 (30.6%)	0.44

Index VTE Event Type, n(%)						
DVT only	5,242 (42.5%)	3,216 (43.4%)	1.85	5,294 (42.9%)	3,183 (43.0%)	0.10
PE with or without DVT	7,098 (57.5%)	4,195 (56.6%)	1.85	7,046 (57.1%)	4,228 (57.0%)	0.10
Index VTE Etiology, n(%)						
Provoked	8,305 (67.3%)	4,673 (63.1%)	8.92	8,108 (65.7%)	4,875 (65.8%)	0.14
Unprovoked	4,035 (32.7%)	2,738 (36.9%)	8.92	4,232 (34.3%)	2,536 (34.2%)	0.14
Deyo-Charlson Comorbidity Index, Mean (SD)	2.9 (2.6)	2.8 (2.6)	3.73	2.9 (2.6)	2.9 (2.6)	0.45
Baseline Comorbidity, n(%)						
Alcohol abuse	269 (2.2%)	161 (2.2%)	0.05	272 (2.2%)	169 (2.3%)	0.49
Anemia	4,253 (34.5%)	2,253 (30.4%)	8.69	4,062 (32.9%)	2,440 (32.9%)	0.03
Central venous Catheter	1,677 (13.6%)	816 (11.0%)	7.86	1,561 (12.7%)	939 (12.7%)	0.03
Hematologic disorders associated with bleeding	1,165 (9.4%)	545 (7.4%)	7.53	1,068 (8.7%)	643 (8.7%)	0.06
Ischemic heart/ coronary artery disease	3,557 (28.8%)	2,148 (29.0%)	0.35	3,576 (29.0%)	2,157 (29.1%)	0.26
Dyspepsia or stomach discomfort	3,086 (25.0%)	1,846 (24.9%)	0.23	3,090 (25.0%)	1,869 (25.2%)	0.40
Hyperlipidemia	6,773 (54.9%)	4,182 (56.4%)	3.11	6,854 (55.5%)	4,122 (55.6%)	0.15
Pneumonia	2,011 (16.3%)	1,257 (17.0%)	1.79	2,043 (16.6%)	1,225 (16.5%)	0.10
Rheumatologic disease	604 (4.9%)	364 (4.9%)	0.08	606 (4.9%)	366 (4.9%)	0.11
Sleep apnea	4,715 (38.2%)	2,761 (37.3%)	1.97	4,674 (37.9%)	2,804 (37.8%)	0.09
Spinal cord injury	27 (0.2%)	21 (0.3%)	1.29	30 (0.2%)	18 (0.2%)	0.14
Thrombophilia	554 (4.5%)	294 (4.0%)	2.60	532 (4.3%)	326 (4.4%)	0.41
Varicose Veins	675 (5.5%)	457 (6.2%)	2.98	707 (5.7%)	427 (5.8%)	0.11
Hypertension	10,233 (82.9%)	6,131 (82.7%)	0.52	10,235 (82.9%)	6,155 (83.1%)	0.31
Non-ESRD Renal Disease	2,431 (19.7%)	1,415 (19.1%)	1.53	2,416 (19.6%)	1,463 (19.7%)	0.41
End Stage Renal Disease	409 (3.3%)	132 (1.8%)	9.74	339 (2.7%)	207 (2.8%)	0.26
Inflammatory Bowel Disease	176 (1.4%)	101 (1.4%)	0.54	173 (1.4%)	102 (1.4%)	0.21
Baseline bleed	2,961 (24.0%)	1,460 (19.7%)	10.41	2,767 (22.4%)	1,665 (22.5%)	0.10
Recent History of Falls, n(%)	741 (6.0%)	436 (5.9%)	0.51	737 (6.0%)	443 (6.0%)	0.05
Fracture/trauma involving Lower Extremities, n(%)	1,800 (14.6%)	1,126 (15.2%)	1.70	1,824 (14.8%)	1,092 (14.7%)	0.13
Selected Surgeries, n(%)	3,912 (31.7%)	2,292 (30.9%)	1.67	3,888 (31.5%)	2,332 (31.5%)	0.08
Baseline Medication Use, n(%)						
Antiarrhythmic	1,453 (11.8%)	862 (11.6%)	0.45	1,446 (11.7%)	868 (11.7%)	0.04
Statins	5,277 (42.8%)	3,188 (43.0%)	0.51	5,306 (43.0%)	3,205 (43.2%)	0.50

Anti-platelets	895 (7.3%)	568 (7.7%)	1.57	917 (7.4%)	548 (7.4%)	0.15
Aromatase Inhibitors	25 (0.2%)	17 (0.2%)	0.58	27 (0.2%)	16 (0.2%)	0.12
Beta Blockers	5,001 (40.5%)	2,985 (40.3%)	0.51	4,993 (40.5%)	3,006 (40.6%)	0.22
Gastroprotective Agents	4,022 (32.6%)	2,388 (32.2%)	0.79	4,009 (32.5%)	2,408 (32.5%)	0.02
SERMS	37 (0.3%)	27 (0.4%)	1.12	41 (0.3%)	25 (0.3%)	0.18
NSAIDs	3,983 (32.3%)	2,682 (36.2%)	8.25	4,156 (33.7%)	2,490 (33.6%)	0.16
Hormone Therapy (estrogen)	610 (4.9%)	446 (6.0%)	4.72	651 (5.3%)	385 (5.2%)	0.36

DVT: Deep vein thrombosis; ESRD: End stage renal disease; IPTW: Inverse probability treatment weighting; NSAIDs: Nonsteroidal anti-inflammatory drug; SERMS: Selective estrogen receptor modulator; PE: Pulmonary embolism; SD: Standard deviation; STD: Standardized differences; VTE: Venous thromboembolism

[a]Std Difference=100*|actual std diff|. Std Difference greater than 10 is considered significant.

[b] After applying weights, the values for age category were not whole numbers; therefore, due to rounding the sum of patients does not equal 100%

[c] After applying weights, the values for region category were not whole numbers; therefore, due to rounding the sum of patients does not equal 100%

Supplemental Table 4. Post IPTW Incidence Rate of Recurrent VTE, Major Bleeding, and CRNM Bleeding among Obese and Morbid Obese VTE Patients who initiated Apixaban vs Warfarin

		Warfarin Cohort (Reference)	Apixaban Cohort
**Obese Patients**			
Sample Size		25,602	17,493
Recurrent VTE		638 (2.5%)	317 (1.8%)
	Incidence rate per 100 person-years (95% CI)	7.3 (6.8,7.9)	5.6 (5.0,6.2)
	Number of person-years	8,735.4	5,688.5
Major Bleeding		546 (2.1%)	265 (1.5%)
	Incidence rate per 100 person-years (95% CI)	6.2 (5.7,6.8)	4.6 (4.1,5.2)
	Number of person-years	8,762.4	5,706.6
CRNM Bleeding		2,843 (11.1%)	1,579 (9.0%)
	Incidence rate per 100 person-years (95% CI)	34.8 (33.5,36.1)	29.2 (27.8, 30.7)
	Number of person-years	8,181.5	5,397.7
**Morbid Obese Patients**			
Sample Size		12,340	7,411
Recurrent VTE		337 (2.7%)	130 (1.8%)
	Incidence rate per 100 person-years (95% CI)	8.0 (7.2,8.9)	5.4 (4.5,6.4)
	Number of person-years	4,212.2	2,412.9
Major Bleeding		268 (2.2%)	107 (1.4%)
	Incidence rate per 100 person-years (95% CI)	6.3 (5.6,7.2)	4.4 (3.6,5.33)
	Number of person-years	4,230.8	2,418.8
CRNM Bleeding		1,495 (12.1%)	672 (9.1%)
	Incidence rate per 100 person-years (95% CI)	38.2 (36.3,40.1)	29.3 (27.2,31.6)
	Number of person-years	3,919.5	2,290.4

CI: Confidence Interval; CRNM: Clinically Relevant Non-Major (Bleeding); VTE: Venous Thromboembolism
